# Relationship between exercise beliefs, happiness, quality of life and sociodemographic characteristics in pregnant women in Eastern Turkey: a cross-sectional study

**DOI:** 10.1136/bmjopen-2025-101297

**Published:** 2025-12-18

**Authors:** Burhan Taşkaya, Necmettin Çiftci, Mustafa Durmuş, Fatih Şahin, Metin Yildiz, Hilal Yıldırım

**Affiliations:** 1 Department of Therapy and Rehabilitation, Muş Alparslan Üniversitesi, Muş, Turkey; 2 Department of Nursing, Muş Alparslan Üniversitesi, Muş, Turkey; 3 Department of Midwifery, Sakarya University, Sakarya, Turkey; 4 Department of Public Health Nursing, Inonu University, Malatya, Turkey

**Keywords:** Primary Prevention, Health, Attitude, Cross-Sectional Studies, Exercise

## Abstract

**Abstract:**

**Objective:**

This study was conducted to examine the relationship between exercise health beliefs, happiness and quality of life among pregnant women, and to evaluate how sociodemographic characteristics are associated with these variables.

**Design:**

The study was conducted using a cross-sectional descriptive study design. Data were collected through face-to-face interviews using a convenience sampling approach. ‘Personal Information Form’, ‘Exercise Health Belief Model Scale’, ‘Oxford Happiness Scale Short Form’ and ‘Short Form (SF-12) Quality of Life Scale’ were used to collect data. Descriptive statistics (mean, SD, percentage), Pearson correlation analysis and simple linear regression were applied.

**Setting:**

The study was conducted at Family Health Centres located in a provincial centre in Eastern Turkey.

**Participants:**

A total of 1090 pregnant women who visited the Family Health Centres participated in the study.

**Results:**

The mean score for the Exercise Health Belief Model Scale was 113.98±20.49, the mean score for the Oxford Happiness Scale Short Form was 18.45±4.05 and the mean score for the SF-12 Quality of Life Scale was 85.34±10.29. A positive and moderately significant correlation was found between exercise health beliefs, happiness and quality of life (r=0.31–0.38, p<0.01). Regression analyses showed that exercise health beliefs were significantly associated with both happiness (R²=0.097, p<0.01) and quality of life (R²=0.147, p<0.01).

**Conclusions:**

Higher levels of exercise health beliefs were associated with increased happiness and quality of life in pregnant women. These findings suggest that nursing interventions aimed at strengthening exercise beliefs may contribute to maternal well-being during pregnancy.

**Strengths and limitations of this study**

- A substantial sample size enhanced the statistical power of the analysis.
- Standardised and validated scales were used to ensure measurement reliability.
- The cross-sectional design constrains the capacity to establish causal relationships.
- The restriction to a single provincial centre constrains the generalisability of the results.

## Introduction

 Pregnancy is a sensitive period in which physical, social and psychological changes occur that affect a woman’s life in different ways. This period is an important time when pregnant women should exhibit positive health behaviours for their own health and that of their babies.^1^ In this regard, understanding the underlying factors that shape women’s health-related behaviours during pregnancy becomes crucial.^2^ However, despite the known benefits of a healthy lifestyle during pregnancy, many pregnant women do not engage in recommended levels of health-promoting behaviours such as regular exercise.^3 4^ This situation highlights the need to investigate the psychological and perceptual factors that may be associated with such behaviours.

Health beliefs shape individuals’ actions and attitudes towards maintaining their health or managing their disease. As a result of individuals’ beliefs about health, their health behaviours are reinforced. In this context, whether an individual’s health is affected positively or negatively depends on health beliefs.^2^ Women may be more receptive to adopting positive lifestyle behaviours during pregnancy, given their concern for the baby’s health.^5^ In particular, beliefs regarding the benefits, risks and barriers of exercise may play a key role in determining whether pregnant women engage in physical activity.^6 7^ Nevertheless, it remains unclear how exercise-related health beliefs are associated with broader indicators of psychological well-being and daily functioning in pregnant women.

Exercise is seen as an essential element of a healthy lifestyle that contributes to the prevention and treatment of various diseases.^8^ Pregnant women, in particular, often seek ways to remain active and maintain their health. Pregnant women are often looking for ways to stay active and healthy.^9^ In this context, exercise stands out as one of the most effective strategies for translating health beliefs into tangible health-promoting behaviours.^10^ As a result, exercise has become a key factor in the maintenance and termination of pregnancy.^11^ Additionally, exercise contributes significantly to the improvement of the health of the mother and fetus during pregnancy.^12^ Exercise during pregnancy has many advantages such as regulating organ systems, controlling the woman’s weight, strengthening the muscular activities necessary for childbirth and faster postpartum recovery.^8^ It has been reported in the literature that exercise has positive effects on all tissues and systems during pregnancy and at the time of delivery.^8 11^ Despite these well-documented benefits, psychological determinants such as happiness and quality of life, which may support or hinder engagement in exercise, have not been sufficiently integrated into a unified framework.

Happiness is an individual’s positive evaluation of a subjective experience. In other words, it is an important factor that increases the coping power of individuals against life events.^13^ In addition to improving health, happiness is also an important factor in maintaining health.^14^ Considering that pregnancy is already a period of vulnerability, the role of happiness becomes even more central in supporting both psychological well-being and health behaviours.^15^ Happiness during pregnancy positively affects healthy behaviours of pregnant women and is associated with increased life satisfaction, quality of life and positive emotions.^16 17^ From a public health perspective, determining the factors affecting happiness during pregnancy is critical for prenatal health policies and practices.^18^

High quality of life is directly associated with positive emotions such as happiness in individuals’ lives and healthy lifestyle behaviours.^19^ Thus, quality of life is not an isolated concept but one that is closely intertwined with happiness and health behaviours, including exercise.^19 20^ Quality of life is seen as an important concept with all its dimensions for the healthy completion and maintenance of the pregnancy process for the mother and baby.^12^ Although pregnancy is a normal part of the human life cycle, it causes some changes in women’s lives, and these changes can affect quality of life and happiness.^21^ However, evidence regarding how exercise beliefs, happiness and quality of life coexist and interact during pregnancy is limited.

Research on the relationships between these three constructs and their connections to sociodemographic characteristics in pregnant women is limited. Therefore, this study aimed to determine the relationship between exercise beliefs, happiness and quality of life in pregnant women, and to examine the relationship between demographic characteristics and these variables.

### Research questions

RQ1: Is there a relationship between exercise beliefs, happiness and quality of life in pregnant women?

RQ2: Is there a relationship between happiness and quality of life in pregnant women?

RQ3: Is there a relationship between demographic characteristics and exercise beliefs, happiness and quality of life in pregnant women?

## Methods

### Study design and setting

This study was conducted using a correlational cross-sectional descriptive design to examine the relationship between exercise beliefs, happiness and quality of life in pregnant women. The study was conducted between 22 August 2022 and 30 May 2023. The research was conducted in Family Health Centres (FHCs) located in a provincial centre in the eastern region of Turkey, where routine antenatal follow-up services are provided. The city hosts several public health institutions, including a state hospital, a private medical centre and primary healthcare centres. For this study, data were collected in FHCs that provide maternal and child health services and where pregnant women frequently attend for follow-up visits.

### Population and sample

The population of this study consisted of pregnant women who applied to FHCs in a provincial centre located in Eastern Turkey. The minimum sample size was calculated to be 384 using the unknown population sampling formula (n=t²·p· q/d ²), with a 95% CI (d=0.05), t=1.96, p=0.5 and q=0.5. In total, 1090 pregnant women were included in this study. Data were collected through face-to-face interviews, using convenience sampling. In the post hoc power analysis conducted based on the results obtained from 1090 participants, the statistical power of the study was calculated as 99% at a 95% confidence level with a medium effect size.^22^ The study was reported in accordance with the Strengthening the Reporting of Observational Studies in Epidemiology guidelines.^23^

### Inclusion criteria

All pregnant women who agreed to participate in the study, were able to understand and fill out the research forms, were over 18 years of age and volunteered to participate in the study were included.

### Exclusion criteria

Data collection forms that left the study data incomplete or blank were not included in the study.

### Data collection tools

The data were gathered using the personal information form, which was developed by the researchers, in conjunction with three validated instruments from the literature: the Exercise Health Belief Model Scale (EHBMS), Oxford Happiness Questionnaire Short Form (OHS-S) and Short Form (SF-12) Quality of Life Scale.

### Personal information form

The personal information form prepared by the researchers consisted of questions such as (age, family type, education level and income status).

### Exercise Health Belief Model Scale

The EHBMS was developed by Esparza-Del Villar *et al*^24^ The validity and reliability of the Turkish version was conducted by Çiftci and Kadioğlu.^25^ The scale consists of 32 items and is a 5-point Likert-type scale. Questions 1–26 are graded as not at all (1), a little (2), neither a little nor a lot (3), quite a lot (4) and very much (5). From questions 27 to 32, it is graded as I never think (1), I do not think (2), I think (3), I mostly think (4) and I always think (5). The scale consists of five subdimensions (general health, perception of seriousness, perception of benefit, perception of motivation and perception of sensitivity), with no reverse items. The highest score obtained from the scale was 160, and the lowest score was 32. In the evaluation of the scale, an increase in the score indicated an increase in the level of exercise belief. The Cronbach’s alpha internal consistency coefficient of the scale adapted to Turkish was found to be 0.87.^25^ In this study, Cronbach’s alpha internal consistency coefficient was 0.94.

### Oxford Happiness Scale Short Form

The OHS-S was developed by Hills and Argyle.^26^ The scale was adapted into Turkish by Doğan and Akıncı Çötok.^27^ The Turkish adapted OLS-K consists of 7 items and is a 5-point Likert-type scale. Items 1 and 7 were reverse coded. This scale consists of one dimension. A high score indicates that the happiness level of an individual is high. The Cronbach’s alpha internal consistency coefficient of the scale adapted to Turkish was found to be 0.74.^27^ In this study, Cronbach’s alpha internal consistency coefficient was 0.70.

### SF-12 Quality of Life Scale

The SF-12 Quality of Life Scale was developed by Ware *et al.* SF-12 was created by selecting and scoring 12 items, including the Physical Component Summary (PCS) and Mental Component Summary (MCS) of SF-36.^28^ The SF-12 was adapted into Turkish by Soylu and Kütük.^29^ SF-12, which was developed to measure general health status and health-related quality of life, consists of 8 subdimensions and 12 items including Physical Functioning (2 items), Physical Role (2 items), Body Pain (1 item), General Health (1 item), Energy (1 item), Social Functioning (1 item), Emotional Role (2 items) and Mental Health (2 items). In the scale consisting of a total of 12 items, a total of four items, items 1, 8, 9 and 10, were reverse coded. Items related to physical and emotional roles were answered as yes or no, while the other items had Likert-type options between 3 and 6. The SF-12 provides a summary of the assessment of both physical and mental health. The PCS-12 score was obtained from the general health, physical functioning, physical role and body pain subscales, while the MCS-12 score was obtained from the social functioning, emotional role, mental health and energy subscales. Both FCI-12 and MCI-12 scores range from 0 to 100, with higher scores representing better quality of life.^29^ The Cronbach’s alpha internal consistency coefficient of the FCI subdimension was found to be 0.73, and the Cronbach’s alpha internal consistency coefficient of the MCI subdimension was found to be 0.72. In this study, the Cronbach’s alpha internal consistency coefficients of the subdimensions were 0.74 and 0.70, respectively.

### Data collection

Necessary institutional permission was obtained before research data were collected. Data were collected face-to-face by researchers. Pregnant women were informed about the purpose of the study, that their answers to the questionnaires would be kept anonymous and confidential, and that the collected data would be used only for academic research. In alignment with the data privacy principles, data collection from each participant was conducted after obtaining informed consent. Participants were asked to approve this form before starting the study. It took approximately 15–20 min for the participants to answer the survey questions.

### Data analysis

SPSS V.24 was used for data analysis. Descriptive statistics (frequency, percentage, mean and SD) were used to summarise the data. The strength of the correlation coefficients was interpreted as follows: 0.99–0.60=high, 0.59–0.30=moderate, 0.29–0.01=low and 0=no correlation.^30^ It has been suggested that when the sample size is greater than 50 and the skewness and kurtosis values range between –1.5 and +1.5, the data can be considered normally distributed.^31^ In this study, the independent samples t-test was used for the two groups based on the normal distribution condition. When there were more than two groups, one-way analysis of variance (ANOVA) was used. Pearson’s correlation analysis was conducted to examine the relationships among the variables, while a simple linear regression analysis was applied to determine predictive associations. The reliability of the scales was assessed using Cronbach’s alpha coefficient. For statistical comparison, all categorical sociodemographic variables were coded numerically. Family type (0=extended, 1=nuclear), education level (1=illiterate, 2=literate, 3=primary, 4=secondary, 5=higher education), spouse’s education level (1–5 coded similarly), income status (1=low, 2=middle, 3=high), chronic disease status (0=no, 1=yes) and employment status (0=unemployed, 1=employed) were coded to allow for group comparisons by using t-tests and ANOVA. Age was considered a continuous variable. These coding procedures enabled statistical tests to be used in this study.

### Patient and public involvement

Patients and/or the public were not involved in the design, conduct, reporting or dissemination of this research. 

## Findings

Descriptive characteristics of the pregnant women included in this study are shown in (table 1).

**Table 1:** Comparison of demographic characteristics of pregnant women and mean scale scores (N=1090)

Feature	X±SD	Min-Max	EHBMS		OHS-S		SF-12	
			Test and p value		Test and p value		Test and p value	
Age	28.56±5.72	18–43	r:0.45 P:0.141		r:0.008 P:0.795		r:−0.12 P:0.695	
	**n**	**%**	**Ort.±SS**	**Test and p value**	**Ort.±SS**	**Test and p value**	**Ort.±SS**	**Test and p value**
Family type								
Nuclear family	813	74.6	116.15±17.41	**†T:4.708**	18.78±3.69	**†T:4.925**	85.62±7.71	†T:1.220
Extended family	277	25.4	107.57±26.97	P:0.00**	17.46±4.62	P:0.00**	84.53±13.80	P:0.170
Education level								
Illiterate	136	12.4	108.66±16.21		17.77±3.79		85.89±7.15	
Literate	47	4.3	111.33±29.22	**‡F:4.550**	16.00±4.59	**‡F:5.530**	80.23±14.13	**‡F:3.717**
Primary education	247	22.6	114.00±21.75	P=0.001**	18.63±3.92	P=0.000**	86.59±10.72	P=0.005**
Secondary education	430	39.4	113.78±19.72	**5–4,3”**	18.70±3.88	**1–2,3,4”**	85.11±10.39	**1–5”**
Higher education	230	21.1	117.92±20.73		18.62±4.08		85.06±9.95	
Spouse’s education level								
Illiterate	53	4.8	109.22±18.40		16.92±4.38		87.85±7.31	
Literate	30	2.7	107.88±20.73	**‡F:5.287**	17.50±2.78	**‡F:3.119**	87.10±9.60	**‡F:2.524**
Primary education	171	15.6	109.57±23.14	P=0.000**	18.74±4.07	P=0.014*	84.99±11.48	p=0.039*
Secondary education	415	38	113.96±22.87	**4–2”**	18.30±4.36	**4–5”**	84.34±11.55	**3–5”**
Higher education	421	38.6	116.77±16.55		18.73±3.52		86.05±8.46	
Income status								
Low	195	17.9	110.67±19.38	**‡F:7.240**	17.09±3.64	**‡F:13.927**	84.64±9.61	‡F:2.663
Middle	777	71.3	115.47±20.52	P=0.001**	18.78±3.94	P=0.000**	85.77±10.29	p=0.070
High	118	10.8	109.72±20.08	**2–1,3”**	18.49±4.34	**1–2,3”**	83.70±10.85	
Chronic disease								
Yes	227	20.8	116.57±16.21	**†T:2.157**	16.92±4.69	**†T:−6.531**	84.40±10.11	†T:−1.558
No	863	79.2	113.29±21.57	P:0.001**	18.85±3.69	P:0.000**	85.59±10.28	P:0.647
Employment status								
Yes	190	17.4	119.49±13.53	**†T:4.147**	17.39±5.29	**†T:−4.016**	82.48±9.35	**†T:−4.300**
No	900	82.6	112.79±21.63	P:0.000**	18.68±3.62	P:0.000**	85.96±10.34	P:0.000**

Ort.±SS” refers to Mean ± Standard Deviation

Bold values indicate statistically significant group differences. (**p<0.01. *p<0.05.) a = Independent-samples t-test, b = One-way ANOVA, ” = Post hoc (Tamhane test).

* p<0.05; **p<0.01.

† Independent-Sample t-test.

‡ One-way ANOVA.

ANOVA, analysis of variance; EHBMS, Exercise Health Belief Model Scale; Mean ± SD, Mean ± standard deviation; OHS-S, Oxford Happiness Scale Short Form.

The mean age of the pregnant women was 28.56±5.72 years, 74.6% lived in nuclear families, 39.4% had secondary education, 38.6% of their spouses had higher education, 71.3% had moderate income, 79.2% had no chronic disease, 63.5% had never had an abortion and 82.6% were employed. A statistically significant difference was found between the mean total scores of the EHBMS and OHS-S and family type, education level, spouse’s education level, income level, presence of chronic disease and employment status (p<0.01, p<0.05) (table 1). A statistically significant difference was found between the mean scores of the total quality of life of pregnant women and their educational level, spouse’s educational level and employment status (p<0.01, p<0.05) (table 1).

When the minimum-maximum mean scores obtained from the scales were examined: the EHBMS (113.98±20.49), General Health Subscale (14.56±3.60), Subscale (24.17±5.07), Subscale (36.73±8.58), Subscale (22.28±5.23) and Subscale (16.27±5.01). The mean scores of the OHS (18.45±4.05), SF-12 Quality of Life Scale (85.34±10.29), Physical Component Subscale (40.16±6.28) and Mental Component Subscale (45.18±7.69) were determined (table 2).

**Table 2:** Distribution of min-max and mean scores of pregnant women in exercise belief, happiness and quality of life scales

Scales	Min	Max	X±SD
Exercise Health Belief Model Scale	45	160	113.98±20.49
General Health Subscale	4	20	14.56±3.60
Perception of Seriousness Subscale	6	30	24.17±5.07
Benefit Perception Subscale	10	50	36.73±8.58
Motivation Subscale	6	30	22.28±5.23
Sensitivity Perception Subscale	6	30	16.27±5.01
Oxford Happiness Scale Short Form	7	33	18.45±4.05
SF-12 Quality of Life Scale	59.83	100	85.34±10.29
Physical Component Subscale	25.46	56.49	40.16±6.28
Mental Component Subscale	14.39	68.70	45.18±7.69

SF-12, Short Form 12.

In this study, a positive and statistically significant correlation was found between OHS-S and SF-12 total scores (r=0.323**, p<0.01) and Physical Component Subscale (r=0.234**, p<0.01) and the Mental Component Subscale (r=0.242**, p<0.01) (table 3).

**Table 3:** The relationship between exercise beliefs, quality of life and happiness levels of pregnant women

Scales			(OHS-S) Total	SF-12 Quality of Life Scale			Exercise Health Belief Model Scale (EHBMS)					
				SF-12 Total	Physical Component	Mental Component	EHBMS Total	General Health	Seriousness	Benefit	Motivation	Sensitivity
Oxford Happiness Scale Short Form (OHS-S)		r	1	0.323^**^	0.234^**^	0.242^**^	0.312^**^	0.367^**^	0.330^**^	0.180^**^	0.210^**^	0.143^**^
		p		**0.000**	**0.000**	**0.000**	**0.000**	**0.000**	**0.000**	**0.000**	**0.000**	**0.000**
SF-12 Quality of Life Scale	SF-12 Total	r	0.323^**^	1	0.666^**^	0.793^**^	0.383^**^	0.281^**^	0.370^**^	0.299^**^	0.314^**^	0.146^**^
		p	**0.000**		**0.000**	**0.000**	**0.000**	**0.000**	**0.000**	**0.000**	**0.000**	**0.000**
	Physical Component	r	0.234^**^	0.666^**^	1	074^**^	0.297^**^	0.217^**^	0.218^**^	0.253^**^	0.277^**^	0.113^**^
		p	**0.000**	**0.000**		**0.019**	**0.000**	**0.000**	**0.000**	**0.000**	**0.000**	**0.000**
	Mental Component	r	0.242^**^	0.793^**^	0.074^*^	1	0.270^**^	0.199^**^	0.317^**^	0.193^**^	0.196^**^	0.103^**^
		p	**0.000**	**0.000**	**0.019**		**0.000**	**0.000**	**0.000**	**0.000**	**0.000**	**0.001**
Exercise Health Belief Model Scale (EHBMS)	EHBMS Total	r	0.312^**^	0.383^**^	0.297^**^	0.270^**^	1	0.720^**^	0.792^**^	0.913^**^	0.891^**^	0.270^**^
		p	**0.000**	**0.000**	**0.000**	**0.000**		**0.000**	**0.000**	**0.000**	**0.000**	**0.000**
	General Health	r	0.367^**^	0.281^**^	0.217^**^	0.199^**^	0.720^**^	1	0.640^**^	0.586^**^	0.594^**^	-0.055^*^
		p	**0.000**	**0.000**	**0.000**	**0.000**	**0.000**		**0.000**	**0.000**	**0.000**	**0.022**
	Perception of Seriousness	r	0.330^**^	0.370^**^	0.218^**^	0.317^**^	0.792^**^	0.640^**^	1	0.632^**^	0.644^**^	0.007
		p	**0.000**	**0.000**	**0.000**	**0.000**	**0.000**	**0.000**		**0.000**	**0.000**	0.723
	Benefit Perception	r	0.180^**^	0.299^**^	0.253^**^	0.193^**^	0.913^**^	0.586^**^	0.632^**^	1	0.864^**^	0.054
		p	**0.000**	**0.000**	**0.000**	**0.000**	**0.000**	**0.000**	**0.000**		**0.000**	0.072
	Motivation	r	0.210^**^	0.314^**^	0.277^**^	0.196^**^	0.891^**^	0.594^**^	0.644^**^	0.864^**^	1	0.037
		p	**0.000**	**0.000**	**0.000**	**0.000**	**0.000**	**0.000**	**0.000**	**0.000**		0.220
	Sensitivity Perception	r	0.143^**^	0.146^**^	0.113^**^	0.103^**^	0.270^**^	-0.055^*^	0.007	0.054	0.037	1
		p	**0.000**	**0.000**	**0.000**	**0.001**	**0.000**	**0.022**	0.723	0.072		0.220

Bold values indicate statistically significant group differences. (**p<0.01. *p<0.05.)

SF-12, Short Form 12.

There was a positive correlation between the OHS-S and EHBMS total scores (r=0.312**, p<0.01) between the OMOS-K and General Health Subscale (r=0.367**, p<0.01), Seriousness Perception Subscale (r=0.330**, p<0.01), Benefit Perception Subscale (r=0.180**, p<0. 01), Motivation Subscale (r=0.210**, p<0.01) and sensitivity perception subscales (r=0.143**, p<0.01) (table 3). SF-12 and EHBMS total scores (r=0.383**, p<0.01), SF-12, and the General Health Subscale (r=0.281**, p<0.01). Perception of Seriousness Subscale (r=0.370**, p<0.01). Perception of Benefit Subscale (r=0.299**, p<0. 01). Motivation Subscale (r=0.314**, p<0.01). and the Sensitivity Perception Subscale (r=0.146**, p<0.01) (table 3). A statistically significant positive correlation was found between the EHBMS and the Physical Component Subscale (r=0.297**, p<0.01) and the Mental Component Subscale (r=0.270**, p<0.01) (table 3). A statistically significant positive correlation was found between the physical component and general health subscales (r=0.217**, p<0.01), Seriousness Perception Subscale (r=0.218**, p<0.01), Benefit Perception Subscale (r=0.253**, p<0.01), Motivation Subscale (r=0.277**, p<0.01) and the Sensitivity Perception Subscale (r=0.113**, p<0.01) (table 3). Mental component and general health subscales (r=0.199**, p<0.01), and Seriousness Perception Subscale (r=0.317**, p<0.01), and Benefit Perception Subscale (r=0.193**, p<0.01), Motivation Subscale (r=0.196**, p<0.01), and the Sensitivity Perception Subscale (r=0103**, p<0.01) (table 3).

According to the results of the regression analysis for the prediction of the relationship, pregnant women’s exercise beliefs have a positive and weakly significant association with happiness and quality of life. The R2 value, which is expressed as the explanatory power of the model, was calculated as follows: 0.097 for happiness (R=0. 312; R2: 0.097; p<0.01), and 0.147 for quality of life (R: 0. 383; R2: 0.147; p<0.01). These values show that the exercise health belief variable explained 9.7% of the happiness variable and 14.7% of the quality of life variable. A 1-point increase in the EHBMS score resulted in a 11,430-point increase in the OHS-S score. This increase was statistically significant (p<0.001). ‘OHS−S=11.430×EHBMS+ε’.

A 1-point increase in the EHBMS score resulted in a 63.403-point increase in the SF-12 score. This indicated a strong and significant effect (p<0.001). ‘SF−12=63.403×EHBMS+ε’ (table 4).

**Table 4:** Regression analysis on the prediction of Exercise Health Belief Model (EHBM), Happiness, Oxford happiness scale short form (OHS-S) and SF-12 Quality of Life Scale.

Independent variable	Dependent variable	B	SE	(β)	t	P value	R	R^2^	F	P value
EHBMS	OHS-S	11.430	0.662	0.312	17.264	0.000	312	0.097	116.196	**0.000**
	SF-12	63.403	1.634	0.383	38.800	0.000	383	0.147	185.898	**0.000**

Bold values indicate statistically significant group differences.

EHBMS, Exercise Health Belief Model Scale; OHS-S, Oxford Happiness Scale Short Form; SF-12, Short Form-12 .

## Discussion

This study aimed to determine the relationship between exercise beliefs, happiness and quality of life among pregnant women. In this section, the findings are discussed in light of the existing literature.

Exercise during pregnancy is a health behaviour that contributes to the prevention of pregnancy-related disorders and is safe for both mother and fetus.^8^ The present study demonstrated that women living in nuclear families reported stronger exercise health beliefs. Similar findings were reported by Sönmez *et al*,^32^ who noted that women in nuclear family structures held more positive attitudes towards exercise knowledge and benefits.^32^ A supportive environment and autonomy within smaller family units may provide pregnant women with greater opportunities to prioritise healthy behaviours.

Educational attainment has also emerged as an important factor associated with exercise beliefs. Women with higher educational levels and spouses with advanced education reported stronger beliefs about exercise. Previous studies support this association, suggesting that greater education enhances awareness of exercise benefits and encourages health-promoting practices.^33 34^ Access to accurate information and higher health literacy may explain these stronger beliefs. In addition, women with a higher income status exhibited more positive beliefs, consistent with the findings of Sönmez *et al*,^32^ who emphasised the role of socioeconomic resources in shaping attitudes towards physical activity. Employment status was positively associated with exercise beliefs, echoing the results of Tan *et al*,^35^ in which working women were found to have greater knowledge about the benefits of exercise.

In this study, a significant relationship was found between happiness levels of pregnant women and economic status, family type, employment status and educational status. The happiness levels of pregnant women with high incomes were found to be higher. In the study conducted by Erato *et al*, it was reported that the happiness levels of pregnant women with high economic status were higher.^18^ It was found that unemployed pregnant women had higher levels of happiness. In a different study, it was reported that there was no significant relationship between the employment status of pregnant women and their happiness.^15^

Quality of life was significantly associated with higher education levels in both women and their spouses, in line with the findings reported by Buldur and Göcen^36^ and Kowalska.^37^ Spousal support, particularly in the psychological and physical domains, appears to play an important role in enhancing women’s quality of life during pregnancy. Employment status was also significantly associated with quality of life, although the findings diverged from earlier studies such as Akpınar and Apay,^38^ where unemployed women reported a lower quality of life. In the present study, the majority of participants had a moderate income, which may have buffered the impact of employment on well-being.

Beyond demographic factors, this study revealed significant correlations between exercise beliefs, happiness and quality of life. These results are consistent with those of Kocak *et al*^39^ and Fathnezhad-Kazemi *et al*,^40^ who reported that women with positive health beliefs about physical activity experienced higher levels of happiness and life satisfaction. Stronger beliefs about the benefits of exercise are likely to facilitate engagement in healthy behaviours, reduce stress and strengthen coping abilities.^41^

Exercise health beliefs were significantly and positively associated with happiness and quality of life. Relaxation, breathing and mental visualisation exercises have been shown to enhance coping skills and improve well-being.^9 42^ Similarly, Cannon *et al*^5^ found that most pregnant women believed that exercise could enhance motivation and happiness. They showed that accurate information and positive perceptions of exercise can encourage continued participation in physical activity, thereby contributing to happiness and physical well-being.^43^

Other studies have further supported this association. Dudonienė and Kuisma^44^ highlighted that positive exercise beliefs during pregnancy increase quality of life, while Bulguroğlu *et al*^45^ and Uzun Aksoy and Gürsoy emphasised that^46^ emphasised that prenatal exercise has been emphasised as playing an important role in enhancing quality of life by reducing pain, improving psychological well-being and facilitating postpartum recovery. Grenier *et al*^11^ argued that counselling interventions focusing on women’s beliefs and values regarding exercise are essential to sustain these positive outcomes.

Finally, a positive and moderately significant relationship was found between quality of life and happiness. Similarly, Lagadec *et al*^47^ similarly reported that women with a higher quality of life experienced pregnancy as a more peaceful and satisfying process. These results highlight the interconnected nature of emotional well-being and perceived quality of life and underscore the importance of comprehensive care.

## Conclusions

This study found that pregnant women with higher exercise health belief scores had higher levels of happiness and better quality of life. A significant association was found between exercise health beliefs and happiness levels of pregnant women and variables such as family type, educational attainment, income status, presence of chronic illness and employment status. Furthermore, a significant correlation was found between the quality of life of the pregnant women and their educational level and employment status. Additionally, a moderately significant positive relationship was identified between exercise health beliefs and both quality of life and happiness. Further investigation using longitudinal or experimental methodologies is necessary to determine the directionality of these relationships more precisely.

## Data availability statement

Data are available on reasonable request.

## References

[1] Boybay Koyuncu S, Dinç A (2022). Health & science 2022 midwifery-1.

[2] Ünsal A (2017). Four basıc concepts of nursıng: human, envıronment, health & dısease, nursıng. Ahi Evran University Journal of Health Sciences.

[3] Santos-Rocha R (2022). Exercise and physical activity during pregnancy and postpartum evidence-based guidelines.

[4] Nascimento SL, Surita FG, Godoy AC (2015). Physical Activity Patterns and Factors Related to Exercise during Pregnancy: A Cross Sectional Study. PLoS One.

[5] Cannon S, Hayman M, Lastella M (2023). Pregnant Women’s Attitudes and Beliefs towards Sleep and Exercise: A Cross-Sectional Survey. Clocks Sleep.

[6] Alyafei A, Easton-Carr R (2024). StatPearls.

[7] Jones CJ, Smith H, Llewellyn C (2014). Evaluating the effectiveness of health belief model interventions in improving adherence: a systematic review. Health Psychol Rev.

[8] Ribeiro MM, Andrade A, Nunes I (2022). Physical exercise in pregnancy: benefits, risks and prescription. J Perinat Med.

[9] Cai C, Vandermeer B, Khurana R (2020). The impact of occupational activities during pregnancy on pregnancy outcomes: a systematic review and metaanalysis. Am J Obstet Gynecol.

[10] Rebelle CC, Jette SL, Mills JM (2022). Physical Activity Beliefs and Behaviors during Pregnancy and their Association with Provider Counseling among Women in the Southern United States. Phys Act Health.

[11] Grenier LN, Atkinson SA, Mottola MF (2021). Be Healthy in Pregnancy: Exploring factors that impact pregnant women’s nutrition and exercise behaviours. Matern Child Nutr.

[12] Sonmezer E, Özköslü MA, Yosmaoğlu HB (2021). The effects of clinical pilates exercises on functional disability, pain, quality of life and lumbopelvic stabilization in pregnant women with low back pain: A randomized controlled study. J Back Musculoskelet Rehabil.

[13] Şahin F, Altun ÖŞ (2022). Concept of Happiness in Schizophrenia. Current Approaches in Psychiatry.

[14] Steptoe A (2019). Happiness and Health. Annu Rev Public Health.

[15] Ali N, Elbarazi I, Al-Maskari F (2023). Happiness and associated factors amongst pregnant women in the United Arab Emirates: The Mutaba’ah Study. PLoS One.

[16] Battulga B, Benjamin MR, Chen H (2021). The Impact of Social Support and Pregnancy on Subjective Well-Being: A Systematic Review. Front Psychol.

[17] Helliwell JF, Huang H, Wang S, Helliwell JF, Layard R, Sachs JD (2019). World happiness report 2019.

[18] Erato G, Shreffler KM, Ciciolla L (2024). Maternal childhood adversity and pregnancy intentions as predictors of pregnancy happiness. J Reprod Infant Psychol.

[19] Kirca N, Şahi̇n Altun Ö, Ejder Apay S (2022). The Relationship between Healthy Lifestyle Behaviors and Quality of Life in Pregnant Women. *Turkiye Klinikleri J Nurs Sci*.

[20] Wójcik M, Aniśko B, Siatkowski I (2024). Quality of life in women with normal pregnancy. Sci Rep.

[21] Arslan S, Okcu G, M Coşkun A (2019). Women’s Perceptıon of Pregnancy and the Affectıng Factors. AHSR.

[22] Cohen J (1988). Statistical power analysis for the behavioral sciences (3rd edition).

[23] Vandenbroucke JP, von Elm E, Altman DG (2007). Strengthening the Reporting of Observational Studies in Epidemiology (STROBE): explanation and elaboration. Epidemiology.

[24] Villar OAE-D, Montañez-Alvarado P, Gutiérrez-Vega M (2017). Factor structure and internal reliability of an exercise health belief model scale in a Mexican population. BMC Public Health.

[25] Çi̇ftci̇ N, Kadioğlu H (2020). Validity and Reliability of the Exercise Health Belief Model Scale. Clin Exp Health Sci.

[26] Hills P, Argyle M (2002). The Oxford Happiness Questionnaire: a compact scale for the measurement of psychological well-being. Pers Individ Dif.

[27] Doğan T, Akıncı Çötok N (2011). Adaptation of the short form of the oxford happiness questionnaire into turkish: a validity and reliability study. Turkish Journal of Psychological Counseling and Guidance.

[28] Ware JE, Keller SD, Kosinski M (1995). SF-12: how to score the SF-12 physical and mental health summary scales.

[29] Soylu C, Kütük B (2022). Reliability and Validity of the Turkish Version of SF-12 Health Survey. *Turk Psikiyatri Derg*.

[30] Büyüköztürk Ş (2002). Factor analysıs: Basıc concepts and usıng to development scale. Educational Management in Theory and Practice.

[31] Tabachnick BG, Fidell LS, Ullman JB (2013). Using multivariate statistics (Sxth ed.).

[32] Sönmez T, Tekgündüz S, Ağduman F (2023). Gebelerin egzersiz tutumlarının belirlenmesi. Anatolian Journal of Health Research.

[33] Hailemariam TT, Gebregiorgis YS, Gebremeskel BF (2020). Physical activity and associated factors among pregnant women in Ethiopia: facility-based cross-sectional study. BMC Pregnancy Childbirth.

[34] Meander L, Lindqvist M, Mogren I (2021). Physical activity and sedentary time during pregnancy and associations with maternal and fetal health outcomes: an epidemiological study. BMC Pregnancy Childbirth.

[35] Tan YR, Tan KH, Dai F (2023). Attitudes and practices of exercise among pregnant mothers in Singapore. Singapur Med J.

[36] Buldur AH, Göcen G (2021). Investigation of religious, spiritual and psychological conditions of women during unintended pregnancy period. JVE.

[37] Kowalska J (2023). The Level of Stress and Anxiety in Pregnant Women Depending on Social Support and Physical Activity. J Clin Med.

[38] Akpınar F, Apay SE (2020). The correlation among pregnancy-related distress and complaints and quality of life during pregnancy. The Journal of Gynecology- Obstetrics and Neonatology.

[39] Kocak MY, Göçen NN, Akin B (2022). The Effect of Listening to the Recitation of the Surah Al-Inshirah on Labor Pain, Anxiety and Comfort in Muslim Women: A Randomized Controlled Study. J Relig Health.

[40] Fathnezhad-Kazemi A, Aslani A, Hajian S (2021). Association between Perceived Social Support and Health-Promoting lifestyle in Pregnant Women: A Cross-Sectional Study. J Caring Sci.

[41] Ayaz-Alkaya S, Yaman-Sözbir Ş, Terzi H (2020). The effect of Health Belief Model-based health education programme on coping with premenstrual syndrome: a randomised controlled trial. Int J Nurs Pract.

[42] Du MC, Ouyang YQ, Nie XF (2019). Effects of physical exercise during pregnancy on maternal and infant outcomes in overweight and obese pregnant women: A meta-analysis. Birth.

[43] Wu S, Feng X, Sun X (2020). Development and evaluation of the health belief model scale for exercise. Int J Nurs Sci.

[44] Dudonienė V, Kuisma R (2023). Women’s Knowledge and Perceptions of the Effect of Exercise during Pregnancy: A Cross-Sectional Study. Int J Environ Res Public Health.

[45] Bulguroğlu Hİ, Bulguroğlu M, Özkul Ç (2023). Investigation of the Effect of Pilates Exercises on Postural Stability and Fear of Birth in Pregnancy. Adnan Menderes Üniversitesi Sağlık Bilimleri Fakültesi Dergisi.

[46] Uzun Aksoy M, Gursoy E, Gazi Universitesi Saglik Bilimleri Fakultesi Hemsirelik Bolumu, Ankara, Turkiye (2021). An Exercise Type in Pregnancy: Prenatal Yoga. J Educ Res Nurs.

[47] Lagadec N, Steinecker M, Kapassi A (2018). Factors influencing the quality of life of pregnant women: a systematic review. BMC Pregnancy Childbirth.

