# Supplementary material for: Relationship between exercise beliefs, happiness, quality of life and sociodemographic characteristics in pregnant women in Eastern Turkey: a cross-sectional study
Source: BMJ Open. 2025 Dec 18;15(12):e101297. doi: 10.1136/bmjopen-2025-101297 (PMC12716570; doi:10.1136/bmjopen-2025-101297)
Supplement: Supplementary file 1 [file bmjopen-2025-101297.reviewer_comments.pdf]

PEER REVIEW HISTORY

BMJ Open publishes all reviews undertaken for accepted manuscripts. Reviewers are asked to complete a checklist review form and are provided with free text boxes to elaborate on their assessment. These free text comments are reproduced below.

ARTICLE DETAILS

Title (Provisional)

The Relationship Between Exercise Beliefs, Happiness, Quality of Life, and Sociodemographic Characteristics in Pregnant Women in Eastern Turkey: A Cross-Sectional Study

Authors

TAŞKAYA, Burhan; ÇİFTCİ, Necmettin; DURMUŞ, Mustafa; ŞAHİN, Fatih; YILDIZ, Metin; Yıldırım, Hilal

---

VERSION 1 - REVIEW

**Reviewer**	**1**
**Name**	**Chergaoui, Samia**
**Affiliation**	**Hassan First University of Settat**
**Date**	**23-May-2025**
**COI**	**None**

The proposed study focuses on the effect of exercise-related beliefs on the happiness and quality of life of pregnant women. However, the introduction mainly consists of a series of definitions of key concepts, without clearly presenting the research problem, study objectives, or its relevance to public health.

It would be valuable to further discuss the scientific relevance of focusing on beliefs, considering that it is the actual practice of physical activity that is recommended for pregnant women to improve their well-being. In this context, a methodological reflection could be considered: would it not be more appropriate to conduct this type of study among married women of reproductive age, in order to assess how their beliefs may influence their future adoption of behaviors beneficial to pregnancy?

Furthermore, the methodology section would benefit from more clarity regarding the statistical tests used and the logical reasoning behind the data analysis. These aspects are currently insufficiently structured.

Regarding inclusion criteria, it would have been advisable to exclude pregnant women with chronic illnesses, as these conditions are known to negatively impact quality of life and psychological well-being, potentially introducing bias into the interpretation of results.

Finally, the results tables should be revised to enhance their readability and presentation, in order to better highlight the data and facilitate analysis.

**Reviewer**	**2**
**Name**	**Winchester , Jason B**
**Affiliation**	**Concordia University, Health and Human Performance**
**Date**	**30-Jul-2025**
**COI**	**None**

Thank you for the opportunity to review this manuscript. Comments are included below, by section, which I hope are helpful in your revision process.

TITLE:

The title of the manuscript does not match the research question as written. The RQ is primarily about demographic influence. but this is not discussed in the title at all.

ABSTRACT:

The abstract does contain some good information, but should be revised. Currently, statistical treatments are not described, but the software package is. Methodology is not discussed in sufficient detail. Data is not included in results, etc.

INTRODUCTION:

The introduction does contain a review of exercise influence on happiness and QOL, but does not seem to significantly address what is currently known about how demographics influence those things in response to exercise. As such, it is impossible to tell what gaps in the literature the authors are intending to address and how their study builds on what is currently known on interrelationship between exercise, happiness, QOL, and demographics.

In addition, in the hypotheses below, we see mention of something referred to as eco-belief. This appears to be the first time this is mentioned, and was not covered in the introduction, nor included in the title, etc.

RESEARCH QUESTION AND HYPOTHESES

There appears to be a mismatch between the RQ and hypotheses. I am unsure what "eco-belief" represents, and this was not included in the RQ.

METHODS:

Insufficient detail on the study design is provided Study cannot be replicated as written. Presentation of methods is, at times, confusing. How were demographics scored in such a way that they could be compared statistically? I'm fine with doing so, but I am not seeing how they are assigned a numerical value in the manuscript.

The statistical treatments are also confusing as written. I am unsure how a Pearson Correlation is being used to compare the results of a regression to variables. Please consider the inclusion of your method for evaluating the strength of association in your correlation analysis. Please include the criterion alpha. It is unclear what is being compared with the simple linear regression.

RESULTS:

The results start off by presenting data on significant differences, but the study design was stated to be correlational. In addition, the methods did not discuss statistical processes for assessing differences between groups. As such, it is unclear to this reviewer how differences were obtained and what they represent.

Results of the regression analysis could be explained more clearly. For example, providing the regression equation and explaining the relationship between unit increase would be very helpful.

DISCUSSION:

Virtually every paragraph begins with "In this study...". Please revise for repetition.

In general, the discussion goes a good job of putting their findings into context of the larger body of literature. Some well-founded speculation on the reason for the relationships that were observed would be appropriate as long as the authors are careful not to over-extend.

VERSION 1 - AUTHOR RESPONSE

Reviewer #1

Comments #1: The proposed study focuses on the effect of exercise-related beliefs on the happiness and quality of life of pregnant women. However, the introduction mainly consists of a series of definitions of key concepts, without clearly presenting the research problem, study objectives, or its relevance to public health.

It would be valuable to further discuss the scientific relevance of focusing on beliefs, considering that it is the actual practice of physical activity that is recommended for pregnant

women to improve their well-being. In this context, a methodological reflection could be considered: would it not be more appropriate to conduct this type of study among married women of reproductive age, in order to assess how their beliefs may influence their future adoption of behaviors beneficial to pregnancy?

Author responses #1: We sincerely thank the reviewer for this constructive feedback. We acknowledge that the initial version of our introduction was more focused on defining concepts and did not sufficiently highlight the research problem, objectives, and public health relevance. Therefore, we revised the entry in line with your suggestions.

Regarding the methodological reflection, we agree that assessing exercise beliefs among women of reproductive age could provide valuable insights into preventive health behaviors. However, we deliberately chose to focus on pregnant women, as pregnancy represents a critical period during which beliefs and behaviors have direct and immediate implications for health outcomes. Additionally, pregnant women may be more receptive to health counseling, making this group particularly relevant for the development of targeted interventions.

Revised Introductıon:

INTRODUCTION

Pregnancy is a sensitive period in which physical, social and psychological changes occur that affect a woman's life in different ways. This period is an important time when pregnant women should exhibit positive health behaviors for their own health and that of their babies (Boybay Koyuncu, 2022). In this regard, understanding the underlying factors that shape women’s health-related behaviors during pregnancy becomes crucial. Health beliefs refer to the thoughts and behaviors of individuals about being healthy or being sick. As a result of individuals' beliefs about health, their health behaviors are reinforced. In this context, whether an individual's

health is affected positively or negatively depends on health beliefs (Ünsal, 2017). Women may be more receptive to adopting positive lifestyle behaviors during pregnancy, given their concern for the baby's health (Cannon et al., 2023).

Exercise is seen as an essential element of a healthy lifestyle that contributes to the prevention and treatment of various diseases (Ribeiro et al., 2022). Pregnant women are often looking for ways to stay active and healthy (Cai et al., 2020). In this context, exercise emerges as one of the most effective strategies to transform health beliefs into concrete health-promoting behaviors (Rebelle et al., 2022). As a result of this search, exercise has emerged as an important element in the healthy maintenance and termination of pregnancy (Grenier et al., 2021). Exercise contributes significantly to the health of mother and fetus during pregnancy (Sonmezer et al., 2021). Exercise during pregnancy has many advantages such as regulating organ systems, controlling the woman's weight, strengthening the muscular activities necessary for childbirth and faster postpartum recovery (Ribeiro et al., 2022). It has been reported in the literature that exercise has positive effects on all tissues and systems during pregnancy and at the time of delivery (Grenier et al., 2021; Ribeiro et al., 2022).

Happiness is an individual's positive evaluation of his/her subjective experience. In other words, it is an important factor that increases the coping power of individuals against life events (Şahin & Altun, 2022). In addition to improving health, happiness is also an important factor in maintaining health (Steptoe, 2019). Considering that pregnancy is already a period of vulnerability, the role of happiness becomes even more central in supporting both psychological well-being and health behaviors (Ali et al., 2023). Happiness during pregnancy positively affects healthy behaviors of pregnant women (Battulga et al., 2021). It also affects the increase in happiness, life satisfaction, quality of life and positive emotions (Helliwell et al., 2019). From a public health perspective, determining the factors affecting happiness during pregnancy is critical for prenatal health policy and practices (Erato et al., 2024).

High quality of life is directly related to positive emotions such as happiness in individuals' lives and healthy lifestyle behaviors (Kırca et al., 2022). Thus, quality of life is not an isolated concept but one that is closely intertwined with happiness and health behaviors, including exercise (Kırca et al., 2022; Wójcik et al., 2024). Quality of life is seen as an important concept with all its dimensions for the healthy completion and maintenance of the pregnancy process for mother and baby (Sonmezer et al., 2021). Although pregnancy is a normal part of the human life cycle, it causes some changes in women's lives. These changes can affect quality of life and happiness (Arslan et al., 2019).

Taken together, the interactions among exercise beliefs, happiness, and quality of life highlight the need for an integrated evaluation of maternal health during pregnancy. Therefore, this study aimed to determine the relationship between exercise beliefs, happiness, and quality of life in pregnant women, while also examining the influence of demographic characteristics on these variables.

Comments #2: Furthermore, the methodology section would benefit from more clarity regarding the statistical tests used and the logical reasoning behind the data analysis. These aspects are currently insufficiently structured.

Author responses #2: We thank the reviewer for this valuable comment. In line with your suggestion, we have expanded the methodology section to provide more clarity on the statistical tests used and the rationale behind our data analysis. Specifically, we have detailed that descriptive statistics (number, percentage, mean, and standard deviation) were first applied to summarize the sociodemographic characteristics of the participants. The normality of data distribution was checked using Skewness and Kurtosis values, and since the sample size was greater than fifty and values were within the acceptable range (-1.5 to +1.5), parametric tests were deemed appropriate. Pearson Correlation Analysis was conducted to examine the

relationships between exercise beliefs, happiness, and quality of life. Simple linear regression analyses were then applied to determine the predictive effects of exercise beliefs on happiness and quality of life. The power of correlation tests was evaluated according to established thresholds (Büyüköztürk, 2002), and the explanatory power of regression models was reported with R^2 values. We have also explained the logical reasoning for choosing these tests, highlighting that they align with the study's aim of examining relationships between variables and the predictive role of exercise beliefs.

Data analysis

The Statistical Package for the Social Sciences (SPSS) version 24 was used for data analysis. Descriptive statistics (frequency, percentage, mean, and standard deviation) were employed to summarize the data. The strength of the correlation coefficients was interpreted as follows: 0.99–0.60 = high, 0.59–0.30 = moderate, 0.29–0.01 = low, and 0 = no correlation (Büyüköztürk, 2002). It has been suggested that when the sample size is greater than 50 and the skewness and kurtosis values range between –1.5 and +1.5, the data can be considered normally distributed (Tabachnick et al., 2013). Pearson correlation analysis was conducted to examine the relationships among the variables, while simple linear regression analysis was applied to determine predictive associations. The reliability of the scales was assessed using Cronbach's alpha coefficients.

Comments #3: Regarding inclusion criteria, it would have been advisable to exclude pregnant women with chronic illnesses, as these conditions are known to negatively impact quality of life and psychological well-being, potentially introducing bias into the interpretation of results.

Author responses #3: We appreciate the reviewer's thoughtful comment regarding the inclusion criteria. We fully acknowledge that chronic illnesses may negatively affect quality of life and psychological well-being, potentially introducing bias. However, we deliberately chose not to exclude pregnant women with chronic illnesses, as our aim was to reflect the real-world

diversity of the pregnant population attending Family Health Centers. Excluding these women might have limited the external validity and generalizability of our findings. To address this concern, we conducted subgroup analyses considering the presence or absence of chronic illnesses. These analyses revealed significant associations between chronic illness and the outcome variables, and we have reported these findings in the results and discussion sections.

Results It was found that there was a significant relationship between exercise health beliefs and happiness levels of pregnant women and family type, education level, income status, presence of chronic disease and employment status.

Discussion

Our study found a significant relationship between the exercise health belief levels of pregnant women, the presence of chronic diseases, and their economic status.

Comments #4: Finally, the results tables should be revised to enhance their readability and presentation, in order to better highlight the data and facilitate analysis.

Author responses #4: We thank the reviewer for this valuable suggestion. In the revised version of the manuscript, the results tables have been reformatted to improve readability and clarity. Specifically, we have adjusted the layout to ensure consistent alignment of variables and statistical values, simplified the table titles and footnotes for easier interpretation, and highlighted significant findings for better visibility. These changes were made to facilitate analysis and allow readers to more clearly understand the key outcomes of the study.

Reviewer #2

Reviewer #2 Comments to the Author

Dr. Jason B Winchester , Concordia University

Comments to the Author:

Thank you for the opportunity to review this manuscript. Comments are included below, by section, which I hope are helpful in your revision process.

Comments #1:

TITLE:

The title of the manuscript does not match the research question as written. The RQ is primarily about demographic influence. but this is not discussed in the title at all.

Author responses #1: Thank you very much for this valuable comment. We agree with your observation that the original title did not sufficiently reflect the research question, especially the aspect regarding the influence of demographic characteristics. In response, we have revised the manuscript title to better align with the study aim and research questions. In addition, we have revised the research questions to ensure that they are fully aligned with the study title and objectives

Revised title:

The Relationship Between Exercise Beliefs, Happiness, and Quality of Life in Pregnant Women

Revised Research Questions:

RQ1: Is there a relationship between demographic characteristics and exercise beliefs, happiness, and quality of life among pregnant women?

RQ2: Is there a relationship between exercise beliefs, happiness, and quality of life in pregnant women?

RQ3: Is there a relationship between happiness and quality of life in pregnant women?

Comments #2:

ABSTRACT:

The abstract does contain some good information, but should be revised. Currently, statistical treatments are not described, but the software package is. Methodology is not discussed in sufficient detail. Data is not included in results, etc.

Author responses #2: We appreciate the reviewer's constructive feedback on the abstract. In the revised version, we have substantially improved the clarity and completeness of the abstract. Specifically, we added a concise description of the methodology, including the study design, sampling technique, and statistical analyses performed. We clarified the statistical treatments used (descriptive statistics, Pearson correlation, and linear regression analyses) instead of only mentioning the software package. Furthermore, we included key quantitative results (mean scores and correlation values) to provide a clearer picture of the findings. Finally, the conclusion was refined to emphasize the main implications of the study for practice and future research.

Revised Research Abstract

Objective This study was conducted to determine the relationship between exercise beliefs, happiness, and quality of life in pregnant women.

Design The study was conducted using a cross-sectional descriptive study design. Data were collected through face-to-face interviews using a probability sampling method. "Personal Information Form", "Exercise Health Belief Model Scale", "Oxford Happiness Scale Short Form" and "SF-12 Quality of Life Scale" were used to collect data. Data were analyzed using

Setting The study was conducted at Family Health Centers (FHCs) located in a provincial center in eastern Turkey.

Participants A total of 1090 pregnant women who visited the Family Health Centers participated in the study.

Results The mean EHBMS score was 113.98±20.49, mean OHS-S score was 18.45±4.05, and mean SF-12 score was 85.34±10.29. A positive, moderately significant correlation was found between exercise health beliefs, happiness, and quality of life ($r = 0.31–0.38$, $p < 0.01$). Regression analyses showed that exercise health beliefs significantly predicted both happiness ($R^2 = 0.097$, $p < 0.01$) and quality of life ($R^2 = 0.147$, $p < 0.01$).

Conclusion Higher levels of exercise health beliefs were associated with increased happiness and quality of life in pregnant women. These findings suggest that nursing interventions aimed at strengthening exercise beliefs may contribute to maternal well-being during pregnancy.

Comments #3:

INTRODUCTION:

The introduction does contain a review of exercise influence on happiness and QOL, but does not seem to significantly address what is currently known about how demographics influence those things in response to exercise. As such, it is impossible to tell what gaps in the literature the authors are intending to address and how their study builds on what is currently known on interrelationship between exercise, happiness, QOL, and demographics.

Author responses #3: We thank the reviewer for this insightful comment. In response, we have revised the introduction to more explicitly address the role of demographic factors in shaping exercise beliefs, happiness, and quality of life.

Comments #4: In addition, in the hypotheses below, we see mention of something referred to as eco-belief. This appears to be the first time this is mentioned, and was not covered in the introduction, nor included in the title, etc.

Author responses #4: We appreciate the reviewer for pointing out this important inconsistency. The reference to "eco-belief" in the hypotheses section was a typographical error. The intended term was "exercise belief," which aligns with the overall focus of the study, the introduction, and the title. We have carefully revised the manuscript to correct this error and ensure consistency in terminology throughout the text.

We apologize for the oversight and thank the reviewer for bringing it to our attention.

Comments #5.

RESEARCH QUESTION AND HYPOTHESES

There appears to be a mismatch between the RQ and hypotheses. I am unsure what "eco-belief" represents, and this was not included in the RQ.

Author responses #5: We thank the reviewer for carefully noting this mismatch. The term "eco-belief" appearing in the research questionss was a typographical error; the intended term was "exercise belief," which is consistent with the research questions, introduction, and title of the study. We have corrected this error to ensure alignment across the research questions, hypotheses, and the overall study framework.

Additionally, we revised the to more clearly reflect and correspond with the stated research questions, thereby strengthening the coherence of the study design.

Revised Research Questions

RQ1: Is there a relationship between demographic characteristics and exercise beliefs, happiness, and quality of life among pregnant women?

RQ2: Is there a relationship between exercise beliefs, happiness, and quality of life in pregnant women?

RQ3: Is there a relationship between happiness and quality of life in pregnant women?

Comments #6:

METHODS:

Insufficient detail on the study design is provided Study cannot be replicated as written. Presentation of methods is, at times, confusing. How were demographics scored in such a way that they could be compared statistically? I'm fine with doing so, but I am not seeing how they are assigned a numerical value in the manuscript.

Author responses #6: Thank you, dear reviewer; the method has been made more systematic. Demographic characteristics were assigned as 1-2-3 according to the survey form and analyzed.

Comments #7: The statistical treatments are also confusing as written. I am unsure how a Pearson Correlation is being used to compare the results of a regression to variables. Please consider the inclusion of your method for evaluating the strength of association in your correlation analysis. Please include the criterion alpha. It is unclear what is being compared with the simple linear regression.

Author responses #7: In the study, correlation analysis was used as a preliminary examination to demonstrate the study's power, as you mentioned. It ensured that regression analysis was performed, as there was neither low power nor excessively high power.

Comments #8: RESULTS:

The results start off by presenting data on significant differences, but the study design was stated to be correlational. In addition, the methods did not discuss statistical processes for assessing differences between groups. As such, it is unclear to this reviewer how differences were obtained and what they represent.

Results of the regression analysis could be explained more clearly. For example, providing the regression equation and explaining the relationship between unit increase would be very helpful.

Author responses #8: ‘’The Relationship Between Exercise Beliefs, Happiness, and Quality of Life in Pregnant Women’’ We organized the title like this. In the study, the independent samples t-test was used for two groups based on the normal distribution condition. When there were more than two groups, the One-Way ANOVA test was used.

A 1-point increase in the EHBMS score results in an 11,430-point increase in the OHS-S value. This increase is statistically significant ($p < .001$). ‘’$OHS-S=11.430\times EHBMS+\varepsilon$’’.

A 1-point increase in the EHBMS score results in a 63.403-point increase in the SF-12 score. This indicates a very strong and significant effect ($p < .001$). ‘’$SF-12=63.403\times EHBMS+\varepsilon$‘’.

Comments #9: Virtually every paragraph begins with "In this study...". Please revise for repetition.

In general, the discussion goes a good job of putting their findings into context of the larger body of literature. Some well-founded speculation on the reason for the relationships that were observed would be appropriate as long as the authors are careful not to over-extend.

Author responses #9: We thank the reviewer for this constructive feedback. In the revised discussion section, we have restructured the paragraphs to avoid repetitive phrasing such as beginning with “In this study…”. We used more varied sentence structures to enhance readability and flow.

Revised Research Discussion

This study aimed to determine the relationship between exercise beliefs and happiness and quality of life in pregnant women. In this section, the findings are discussed in the light of the literature.

Exercise during pregnancy is a health behavior that contributes to the prevention of pregnancy-related disorders and is safe for both mother and fetus (Ribeiro et al., 2022). The present study demonstrated that women living in nuclear families reported stronger exercise health beliefs. Similar findings were reported by Sönmez et al. (Sönmez et al., 2023), who noted that women in nuclear family structures held more positive attitudes toward exercise knowledge and benefits (Sönmez et al., 2023). The supportive environment and autonomy within smaller family units may provide pregnant women with greater opportunities to prioritize healthy behaviors.

Educational attainment also emerged as an important factor influencing exercise beliefs. Women with higher education levels and spouses with advanced education reported stronger beliefs about exercise. Previous studies support this association, suggesting that greater education enhances awareness of exercise benefits and encourages health-promoting practices (Hailemariam et al., 2020; Meander et al., 2021). Access to accurate information and higher health literacy may therefore explain these stronger beliefs. In addition, women with higher income status exhibited more positive beliefs, consistent with the findings of Sönmez et al. (2023), who emphasized the role of socioeconomic resources in shaping attitudes toward physical activity. Employment status also contributed positively to exercise beliefs, echoing the results of Tan et al. (2023), where working women were found to have greater knowledge about the benefits of exercise.

In this study, a significant relationship was found between the happiness levels of pregnant women and economic status, family type, employment status and educational status. It was found that the happiness levels of pregnant women with high income were higher. In the study conducted by Erato et al. (2024), it was reported that the happiness levels of pregnant women with high economic status were higher (Erato et al., 2024). It was found that unemployed pregnant women had higher levels of happiness. In a study different from our

study, it was reported that there was no significant relationship between the employment status of pregnant women and their happiness (Ali et al., 2023).

Quality of life was positively influenced by higher education levels in both women and their spouses, in line with findings reported by Buldur and Göçen (2021) and Kowalska (2023). Spousal support, particularly in the psychological and physical domains, appears to play an important role in enhancing women's quality of life during pregnancy. Employment status also influenced quality of life, although findings diverged from earlier studies such as Akpınar and Apay (2020), where unemployed women reported lower quality of life. In the present study, the majority of participants had moderate income, which may have buffered the impact of employment on well-being.

Beyond demographic influences, this study revealed significant correlations between exercise beliefs, happiness, and quality of life. These results are consistent with Koçak et al. (2022) and Fathnezhad-Kazemi et al. (2021), who reported that women with positive health beliefs about physical activity experienced higher levels of happiness and life satisfaction. Stronger beliefs about the benefits of exercise likely facilitate engagement in healthy behaviors, reduce stress, and strengthen coping abilities (Ayaz-Alkaya et al., 2020).

It was found that exercise health beliefs of pregnant women had a significant positive effect on happiness and quality of life. Relaxation, breathing, and mental visualization exercises have been shown to enhance coping skills and improve well-being (Cai et al., 2020; Du et al., 2019). Similarly, Cannon et al. (2023) found that most pregnant women believed exercise could enhance motivation and happiness. shows that accurate information and positive perceptions of exercise can encourage continued participation in physical activity, thereby contributing to happiness and physical well-being (Wu et al., 2020).

Other studies further support these associations. Dudonienė and Kuisma (2023) highlighted that positive exercise beliefs during pregnancy increase quality of life, while

Bulguroğlu et al. (2023) and Uzun Aksoy & Gürsoy (2021) prenatal exercise has been emphasized as playing an important role in enhancing quality of life by reducing pain, improving psychological well-being, and facilitating postpartum recovery. Grenier et al. (2021) argued that counseling interventions focusing on women's beliefs and values about exercise are essential to sustain these positive outcomes.

Finally, a positive and moderately significant relationship was found between quality of life and happiness. Lagadec et al. (2018) similarly reported that women with higher quality of life experienced pregnancy as a more peaceful and satisfying process. These results highlight the interconnected nature of emotional well-being and perceived quality of life and underscore the importance of comprehensive care.

In summary, by amending our manuscript as outlined above, we have responded to the issues raised by the editor and reviewers. We thank the editor and reviewers for helpful comments. These comments have improved the clarity of our manuscript. We hope you find the amended revision of our manuscript acceptable for publication in BMJ Open, and we look forward to hearing from you in the near future.

VERSION 2 - REVIEW

**Reviewer**	**1**
**Name**	**Chergaoui, Samia**
**Affiliation**	**Hassan First University of Settat**
**Date**	**09-Oct-2025**
**COI**	

Page 1

ABSTRACT

Line 16: I suggest combining the “Design,” “Setting,” and “Participants” sections into a single “Method” section.

Line 39: “Results The mean EHBMS…” – please avoid using abbreviations in the abstract.

Page 2

Line 8: “…in pregnant women.”

STRENGTHS AND LIMITATIONS OF THIS STUDY

Line 10: Please present the strengths of your study first, followed by the limitations.

Line 27: Please add indentation at the beginning of the sentence.

Line 57: “As a result of this search, exercise has emerged…” – which search are you referring to?

Lines 3–6: “Exercise contributes significantly to the health of mother and fetus during pregnancy[8]” – contributes to the improvement of health.

Page 3

Introduction

Please rephrase the introduction to make the research problem clearer. You define each concept separately (exercise beliefs, happiness, and quality of life), but the overall problem statement is not clear.

Please maintain indentation at the beginning of each paragraph throughout the manuscript.

VERSION 2 - AUTHOR RESPONSE

Reviewer #1

Comments #1: ABSTRACT Line 16: I suggest combining the “Design,” “Setting,” and “Participants” sections into a single “Method” section.

Author responses #1: Thank you for your valuable suggestion. As recommended, we have combined the “Design,” “Setting,” and “Participants” subsections into a single “Method” subsection in the Abstract.

Method: This cross-sectional descriptive study was conducted with 1,090 pregnant women who visited Family Health Centers located in a provincial center in eastern Turkey. The data were collected through face-to-face interviews using the convenience sampling method.

“Personal Information Form”, “Exercise Health Belief Model Scale”, “Oxford Happiness Scale Short Form” and “SF-12 Quality of Life Scale” were used to collect data. Descriptive statistics (mean, standard deviation, percentage), Pearson correlation analysis, and simple linear regression were applied.

Comments #2: Line 39: “Results The mean EHBMS…” – please avoid using abbreviations in the abstract.

Author responses #2: Thank you for your valuable comments. In accordance with your recommendation, all abbreviations in the Abstract (EHBMS, OHS-S, SF-12)

The mean score for the Exercise Health Belief Model Scale was 113.98±20.49, the mean score for the Oxford Happiness Scale Short Form was 18.45±4.05, and the mean score for the SF-12 Quality of Life Scale was.

Comments #3: Page 2 Line 8: “…in pregnant women.”

Author responses #3: Thank you for your valuable comments. The relevant sentence has been updated.

Comments #4: STRENGTHS AND LIMITATIONS OF THIS STUDY

Line 10: Please present the strengths of your study first, followed by the limitations.

Author responses #4: Thank you for your valuable comments. As recommended, we have reordered the “Strengths and Limitations” section so that the strengths are presented first, followed by the limitations.

STRENGTHS AND LIMITATIONS OF THIS STUDY

⇒ The substantial sample size enhanced the statistical power of the analysis.

⇒ Standardized and validated scales were used to ensure measurement reliability.

⇒ The cross-sectional design constrains the capacity to establish causal relationships.

⇒ The restriction to a single provincial center constrains the generalizability of the results.

Comments #5: Line 27: Please add indentation at the beginning of the sentence.

Author responses #5: Thank you for your valuable comments. As suggested, indentation has been added at the beginning of the sentence on Line 27 to ensure proper formatting. The manuscript has been updated accordingly.

Comments #6: Line 57: "As a result of this search, exercise has emerged…" – which search are you referring to?

Author responses #6: Thank you for your valuable comments. To avoid ambiguity, the sentence has been rewritten as follows: In this context, exercise stands out as one of the most effective strategies for translating health beliefs into tangible health-promoting behaviors.

Comments #7: Lines 3–6: "Exercise contributes significantly to the health of mother and fetus during pregnancy[8]" – contributes to the improvement of health.

Author responses #7: Thank you for your valuable comments. The sentence has been revised as recommended. The phrase "contributes significantly to the health of the mother and fetus" has been updated to "contributes significantly to the improvement of the health of the mother and fetus during pregnancy."

Comments #8: Page 3

Introduction

Please rephrase the introduction to make the research problem clearer. You define each concept separately (exercise beliefs, happiness, and quality of life), but the overall problem statement is not clear.

Author responses #8: Thank you for your valuable comments. In response to your comment, the Introduction section has been revised to present a clearer and more cohesive research problem.

Revised Introduction:

Pregnancy is a sensitive period in which physical, social and psychological changes occur that affect a woman's life in different ways. This period is an important time when pregnant women should exhibit positive health behaviors for their own health and that of their babies [1]. In this regard, understanding the underlying factors that shape women's health-related behaviors during pregnancy becomes crucial [2]. However, despite the known benefits of a healthy lifestyle during pregnancy, many pregnant women do not engage in recommended levels of health-promoting behaviors such as regular exercise [3, 4]. This situation highlights the need to investigate the psychological and perceptual factors that may influence such behaviors.

Health beliefs shape individuals' actions and attitudes toward maintaining their health or managing disease. As a result of individuals' beliefs about health, their health behaviors are reinforced. In this context, whether an individual's health is affected positively or negatively depends on health beliefs [2]. Women may be more receptive to adopting positive lifestyle behaviors during pregnancy, given their concern for the baby's health [5]. In particular, beliefs regarding the benefits, risks and barriers of exercise may play a key role in determining whether pregnant women engage in physical activity [6, 7]. Nevertheless, how exercise-related health beliefs are associated with broader indicators of psychological well-being and daily functioning in pregnant women remains unclear.

Exercise is seen as an essential element of a healthy lifestyle that contributes to the prevention and treatment of various diseases [8]. Pregnant women, in particular, often seek ways to remain active and maintain their health. Pregnant women are often looking for ways to stay active and healthy [9]. In this context, exercise stands out as one of the most effective strategies

for translating health beliefs into tangible health-promoting behaviors [10]. As a result of this quest, exercise has become a key factor in the healthy maintenance and termination of pregnancy [11]. Additionally, Exercise contributes significantly to the improvement of the health of the mother and fetus during pregnancy [12]. Exercise during pregnancy has many advantages such as regulating organ systems, controlling the woman's weight, strengthening the muscular activities necessary for childbirth and faster postpartum recovery [8]. It has been reported in the literature that exercise has positive effects on all tissues and systems during pregnancy and at the time of delivery [8, 11]. Despite these well-documented benefits, psychological determinants such as happiness and quality of life, which may support or hinder engagement in exercise, have not been sufficiently integrated into a unified framework.

Happiness is an individual's positive evaluation of his/her subjective experience. In other words, it is an important factor that increases the coping power of individuals against life events [13]. In addition to improving health, happiness is also an important factor in maintaining health [14]. Considering that pregnancy is already a period of vulnerability, the role of happiness becomes even more central in supporting both psychological well-being and health behaviors [15]. Happiness during pregnancy positively affects healthy behaviors of pregnant women and is associated with increased life satisfaction, quality of life and positive emotions [16 17]. From a public health perspective, determining the factors affecting happiness during pregnancy is critical for prenatal health policy and practices [18].

High quality of life is directly associated with positive emotions such as happiness in individuals' lives and healthy lifestyle behaviors [19]. Thus, quality of life is not an isolated concept but one that is closely intertwined with happiness and health behaviors, including exercise [19, 20]. Quality of life is seen as an important concept with all its dimensions for the healthy completion and maintenance of the pregnancy process for mother and baby [12]. Although pregnancy is a normal part of the human life cycle, it causes some changes in women's lives, and these

changes can affect quality of life and happiness [21]. However, evidence regarding how exercise beliefs, happiness and quality of life coexist and interact during pregnancy is limited.

Research in the literature on the relationships between these three constructs and their connections to sociodemographic characteristics in pregnant women is quite limited. Therefore, this study aims to determine the relationship between exercise beliefs, happiness and quality of life in pregnant women and to examine the relationship of demographic characteristics with these variables.

Comments #8: Please maintain indentation at the beginning of each paragraph throughout the manuscript.

Author responses #8: As per your suggestion, consistent indentation has been applied at the beginning of all paragraphs throughout the manuscript.

In summary, by amending our manuscript as outlined above, we have responded to the issues raised by the editor and reviewers. We thank the editor and reviewers for helpful comments. These comments have improved the clarity of our manuscript. We hope you find the amended revision of our manuscript acceptable for publication in BMJ Open, and we look forward to hearing from you in the near future.
